# Individual Sensitization Pattern Recognition to Cow’s Milk and Human Milk Differs for Various Clinical Manifestations of Milk Allergy

**DOI:** 10.3390/nu11061331

**Published:** 2019-06-14

**Authors:** Frauke Schocker, Skadi Kull, Christian Schwager, Jochen Behrends, Uta Jappe

**Affiliations:** 1 Division of Clinical and Molecular Allergology, Research Center Borstel - Leibniz Lung Center, Priority Area Asthma and Allergy, Airway Research Center North (ARCN), German Center for Lung Research (DZL), 23845 Borstel, Germany; skull@fz-borstel.de (S.K.); cschwager@fz-borstel.de (C.S.); ujappe@fz-borstel.de (U.J.); 2 Core Facility Fluorescence Cytometry, Research Center Borstel, 23845 Borstel, Germany; jbehrends@fz-borstel.de; 3 Interdisciplinary Allergy Outpatient Clinic, MK III, University of Lübeck, Airway Research Center North (ARCN), German Center for Lung Research (DZL), 23538 Lübeck, Germany

**Keywords:** Cow’s milk allergy (CMA), anaphylaxis, sensitization pattern, cow’s milk allergens, CAP-FEIA (Fluorescence Enzyme Immunoassay), multiplex dot test, basophil activation test (BAT), human breast milk

## Abstract

Cow’s milk allergy (CMA) belongs to one of the most common food allergies in early childhood affecting 2–3% of children under 3 years of age. However, approximately 1% of adults remain allergic to cow’s milk, often showing severe reactions even to traces of milk. In our study, we recruited patients with different clinical manifestations of CMA, including patients with anaphylaxis and less severe symptoms. We assessed the sensitization patterns and allergic responses of these subgroups through different immunological and cell-based methods. Sera of patients were investigated for IgE against whole cow’s milk and its single allergens by CAP- FEIA. In a newly developed in-house multiplex dot assay and a basophil activation test (BAT), cow’s milk allergens, in addition to human breast milk and single allergens from cow’s and human milk were analyzed for IgE recognition and severity of CMA in the included patients. Both the CAP-FEIA routine diagnostic and the multiplex dot test could differentiate CMA with severe from milder allergic reactions by means of the patients’ casein sensitization. The BAT, which mirrors the clinical response in vitro, confirmed that basophils from patients with severe reactions were more reactive to caseins in contrast to the basophils from more moderate CMA patients. By means of this improved component-resolved diagnosis of CMA, individual sensitization patterns could be assessed, also taking sensitization against human milk into consideration.

## 1. Introduction

IgE-mediated cow’s milk allergy (CMA) is a common food allergy affecting 2–3% of young children under 3 years of age, involving the skin, the gastrointestinal tract, the respiratory tract or the cardio-vascular system. A high proportion of young children, approximately 85%, develop a natural tolerance. However, approximately 1% of adults have persisting allergic reactions, often severe and life threatening [1,2]. 

Cow’s milk consists of caseins accounting for approximately 80% and whey proteins accounting for approximately 20% of the total protein content. The major cow milk allergens are the caseins (Bos d 8) yielding α-, ß- and к-caseins. Whey proteins consist of α-lactalbumin (Bos d 4) and ß-lactoglobulin (Bos d 5) among other proteins, e.g., bovine serum albumin (Bos d 6), immunoglobulin (Bos d 7), and lactoferrin [3]. According to the literature, the human IgE response to cow’s milk is highly variable and no single allergen component alone accounts for cow’s milk allergenicity [4]. However, looking for prognostic markers, low casein and ß-lactoglobulin-specific IgE-antibody concentrations were found to be predictive for the resolution of CMA [5], and whole cow’s milk specific IgE above 50 kU_A_/L were associated with persistent CMA when studying a population of children with CMA, in terms of the prognosis for developing tolerance [6]. Garcıa-Ara et al. [7] described caseins to best discriminate between transient and persistent CMA. Ito et al. [8] and D’Urbano et al. [9] confirmed these data. Likewise, recent studies have revealed differences in the IgE- and IgG_4-_ binding to epitopes of caseins, α-lactalbumin and ß-lactoglobulin as predictive markers for oral tolerance or the persistence of CMA, respectively [10]. 

Component-resolved diagnosis (CRD) allows the determination of specific IgE not only against whole cow’s milk, but also against single allergens (α-, ß- and к-caseins, α-lactalbumin, ß-lactoglobulin). Because allergy as such is a highly individual immune response necessitating an individual diagnostic approach, CRD may be helpful for monitoring the degree of severity of CMA. Currently, for routine allergy diagnostic tests, total serum IgE, specific IgE to milk, cheese, and milk from other species as well as single milk allergens are accessible by means of CAP- FEIA (ImmunoCAP). 

In our recent study, we were able to monitor a patient with anaphylactic reactions to traces of cow’s milk by means of allergen-specific IgE and basophil activation test (BAT), before and under omalizumab therapy on the molecular level, also addressing sensitization to human milk proteins [11,12]. 

The objective of the present study is to investigate the combination of diagnostic tests—extended by IgE recognition of whole human milk and human α-lactalbumin—for their capacity to distinguish between different clinical manifestations of CMA. The antibody-based methods such as CAP-FEIA, a newly developed multiplex dot test for the detection of specific IgE (sIgE) against whole milk and single milk allergens, as well as a highly specific and sensitive BAT, were applied in order to differentiate between the degrees of severity of CMA.

## 2. Materials and Methods 

### 2.1. Study Group

Patients were recruited in the allergy outpatient clinics in Borstel and Lübeck and serum samples as well as heparinized whole blood were collected from six patients with a clear history of adverse reactions after milk ingestion in the past, with milk as the only food implicated in the episode. Specific IgE to milk was determined by in vivo and in vitro tests. Total IgE and specific IgE to milk, cheese, milk from other mammalian species and single milk allergens were determined by means of CAP-FEIA (ImmunoCAP, ThermoFisher Scientific, Freiburg, Germany). Challenge tests to milk were not performed in patients reporting life-threatening episodes (ID1–ID2); patient ID3 had a positive challenge in early childhood. The other cases refused the provocation tests as they feared adverse reactions (ID4–ID6). Skin prick tests (SPT)—if not contraindicated—were performed according to the standard procedure, with the prick to prick technique with fresh milk. Histamine dihydrochloride and phosphate-buffered saline (PBS) solution served as positive and negative controls, respectively. The local ethics committee of the University of Lübeck approved this study (approval numbers 10–126, 13–086 and 13–136). Serum and heparinized whole blood samples were additionally obtained from a non-atopic, non-sensitized healthy individual and served as controls. Allergic disease was ruled out by history, determination of total and specific IgE against pollens, house dust mite (HDM) and a panel of food allergens including milk, cheese and milk from other mammalian species. Written informed consents were obtained from all subjects included. 

### 2.2. Dot Blot Test

For the dot blot test, 1 µL aliquots of the allergens (bovine and human milk, bovine α-, ß-,and κ-caseins, bovine ß-lactoglobulin, bovine and human α-lactalbumin) were dotted in a concentration of 1 µg/µl and 2.5 µg/µl onto nitrocellulose membrane strips (Amersham protean 0.45 µm, GE Healthcare, Freiburg, Germany) and dried carefully. Thereafter, the membranes were blocked for 1 h with SynBlock (ImmunoChemistry, Bloomington, MN, USA) (1:1 diluted with TBS-T (tris-buffered saline including 0.05% Tween), pH 7.4). In the next step, sera of milk-allergic patients and serum of a non-allergic control were diluted 1:20 with the exception of ID1 (1:50) in TBS-T and incubated on the strips overnight at room temperature. For detection of bound IgE antibodies, the strips were incubated with a horseradish peroxidase (HRP)-conjugated mouse anti-human IgE (Fc) antibody (Southern Biotech, Birmingham, AL, USA), in a 1:5,000 dilution in TBS-T for 2 h. Immunostaining was performed by means of chemiluminescent Western blot detection using Clarity Western ECL Substrate (Bio-Rad, Hercules, CA, USA). The detection of stained dots was visualized using the ChemiDoc MP System (Bio-Rad, Munich, Germany). 

### 2.3. Basophil Activation Test (BAT)

We performed the BAT according to Schwager et al. [13] using an extensive and specific read-out. BAT was conducted with heparinized whole blood stimulated for 30 minutes at 37 °C. Cow’s milk (purchased from a local food store), human breast milk (recruited in a study on peanut allergen transfer into breast milk [14,15]; ethics approval numbers 08–122 and Az19-114) and the single bovine, as well as human milk allergens bovine α-casein, ß-casein, κ-caseins, bovine ß-lactoglobulin, bovine and human α-lactalbumin (Sigma-Aldrich, Steinheim, Germany) were used in serial 10 fold dilutions (10 µg/mL; 1 µg/mL; 100 ng/mL; 10 ng/mL; 1 ng/mL). 

Formyl-methionyl-leucyl phenylalanine (fMLP; 100 nM, Sigma-Aldrich, Steinheim, Germany), polyclonal goat anti-human IgE (1 mg/mL, abcam, Cambridge, UK), or PBS buffer were chosen as controls. 

Basophils were analyzed using an LSR II flow cytometer. Data analysis was conducted with the FCS Express 6 program. For data visualization, Prism graphics software 6.03 (GraphPad Prism Inc., San Diego, CA, USA) was used. 

## 3. Results

### 3.1. Patients with Different Complexity of Allergic Reactions to Cow’s Milk

We recruited six patients with a history of allergic reactions to milk, revealing different types of clinical reactions as well as degrees of severity. The subjects included had positive responses to milk in SPT with the fresh food, if not contraindicated. 

Of these patients, three patients—two adults (ID1, ID2) and one 9-year-old child (ID3)—suffered from severe allergic reactions to milk. In contrast, three patients (ID4, ID5, ID6) displayed milder allergic symptoms. The clinical data are comprised in Supplemental Table S1, summarizing the patients with severe reactions upon milk contact first (ID1, ID2, ID3), followed by the CMA patients with a milder clinical picture (ID4, ID5, ID6). The patients’ history including comorbidities such as asthma, atopic dermatitis and other sensitizations/allergies are included in Supplemental Table S1.

With the exception of ID1, two blood samples were analyzed of each CMA patient named IDx (first blood sample) and IDx-1 (second blood sample, investigated in parallel to the BAT). 

ID7 was a non-allergic individual serving as a control for the CAP-FEIA, the multiplex dot test and the BAT. 

ID1 has suffered from severe reactions since early childhood, even under cow’s milk avoidance of the nursing mother, and experienced cardioplegia at the age of thirteen in more than one episode after accidental contact with milk. Due to the severe reactions upon accidental contact to milk traces, the patient started an oral immunotherapy (OIT) in 2016, as previously reported in [11].

ID2 had already displayed massive adverse skin reactions as a breast-fed child, also under cow’s milk elimination diet of the nursing mother. In 2008/2009 the patient started a self-guided desensitization with milk products. During that phase, reactions to dietary products occurred primarily exercise-induced with increasing complexity of symptoms. After having avoided milk strictly since 2012, the reactions to accidental contact with milk became more severe (angioedema, flush, urticaria and asthmatic reactions, e.g., after a kiss of her boyfriend who had consumed coffee with milk). Shortly before delivery of her first child and five months thereafter the patient presented to our clinic with the question regarding a potential sensitization to human milk and biological relevance upon contact to her own breast milk while breast feeding.

ID3, a nine-year-old girl, has had a confirmed milk allergy since 2010. The skin and respiratory tract (cough, dyspnea) have been severely affected. Milk and milk products have been strictly avoided since then.

The adult patient ID4 developed reactions to milk at the age of 23. According to the history, the reactions to milk are dose-dependent (different amounts of milk and milk products can be tolerated) and occur exercise-induced (nausea, feeling of swelling throat and troubles with swallowing). 

Patient ID5, 25 years old, first observed allergic reactions to milk at the age of 21 and reported nausea and feeling of tightness in esophagus/trachea following milk ingestion.

Patient ID6 reported CMA at the age of 35. Besides gastrointestinal symptoms the patient reported the feeling of dysphagia and of chest pain (dyspnea?) and has avoided milk strictly since 2018. 

### 3.2. Patients with Severe Reactions

**Figure 1:**
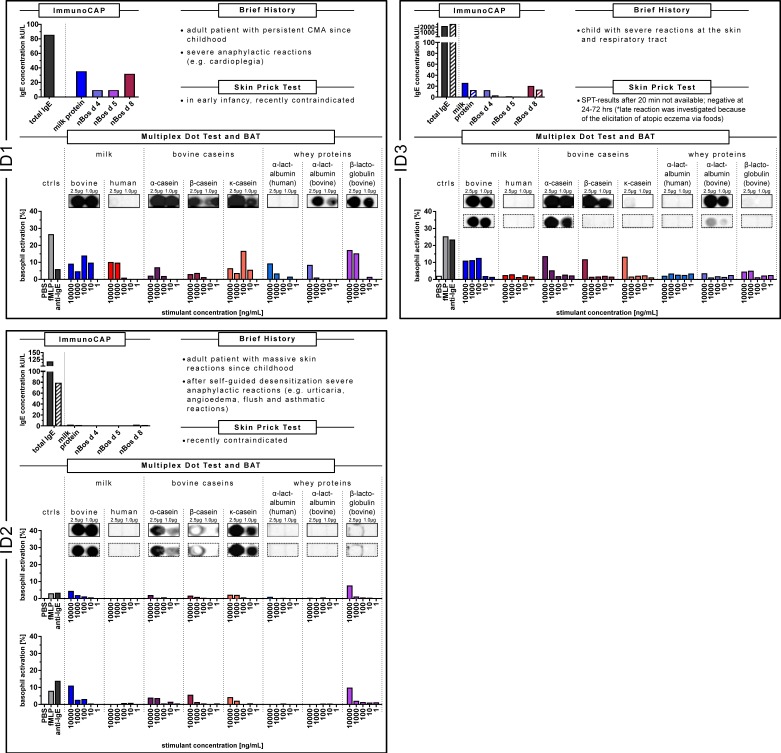
Synopsis of data of the sensitization patterns of ID1, ID2 and ID3 with severe reactions in the ImmunoCAP, multiplex dot test and basophil activation test (BAT). Caseins were associated with this clinical response. CAP data of the cow’s milk allergy (CMA) patients are depicted in a bar graph. The filled bars show the data of the first blood sample, the hatched bars those of the second blood sample. Determination of IgE recognition to whole and single allergens of cow’s milk and human milk in the multiplex dot test. The concentration of the dot-blotted analytes as indicated was 1.0 µg/µL and 2.5 µg/µL, respectively. The IgE recognition of the first blood sample is given in a box with a solid line, of the second blood sample with a box with a dotted line. The BAT was performed with blood of the patients using whole cow’s milk and human milk as well as single bovine and human milk allergens. Percentages represent CD63 positive basophils determined by flow cytometric analysis. Blood sample stimulation was conducted with the analytes as indicated. fMLP, anti-IgE were run as positive controls, PBS as negative control.

Patients ID1, ID2 and ID3 with severe and anaphylactic reactions to cow’s milk depict a similar sensitization pattern in the CAP-FEIA, the multiplex dot test and BAT (comprised in Figure 1). 

As shown in Figure 1 (ID1-ImmunoCAP data, at the top left), the milk anaphylactic patient ID1 displayed a total IgE concentration of 85 IU/mL, a specific IgE against milk protein of 34.9 kU/L and IgE concentrations for caseins of 31.3 kU/L which were higher compared to the whey proteins α-lactalbumin and ß-lactoglobulin of 8.85 kU/L and 8.86 kU/L, respectively. 

In the component-resolved dot test (ID1-Multiplex Dot Test) with IgE recognition not only against bovine but also human milk as well as single milk components (bovine α-, ß-, and κ-caseins, bovine ß-lactoglobulin, bovine and human α-lactalbumin), the patient’s IgE reacted intensively against cow’s milk and the single caseins. IgE reactivity was less pronounced against human milk, however existing. Dotted bovine α-lactalbumin and ß-lactoglobulin also bound antibodies of patient ID1, but less intensively than the caseins. 

Using the BAT as a functional assay to assess the biologic relevance of IgE reactivity, not only cow’s milk but also human milk, and the single human and bovine allergens were tested (ID1-BAT). Patient ID1 was reactive to all extracts and single components: to bovine and human milk, as well as to the caseins and whey proteins. 

Patient ID2 with an increasing complexity of reactions up to anaphylactic symptoms after a self-guided trial of desensitization, displayed a sensitization pattern as shown in Figure 1 (ID2). 

ID2, with total and specific IgE determinations before delivery (ID2; ImmunoCAP data with filled bars), showed a total IgE of 115.7 IU/mL and a low specific IgE against milk protein of only 1.32 kU/L. The casein-specific IgE concentration was 1.27 kU/L in contrast to negative α-lactalbumin- and ß-lactoglobulin-specific IgE. Five months after delivery (ID2–1; ImmunoCAP data with hatched bars), with a total IgE of 78.5 IU/mL; the specific IgE against milk protein was 0.79 kU/L, the casein-specific IgE 0.72 kU/L and the specific IgE against whey proteins were negative. 

Dotted cow’s milk and the single caseins bound antibodies of both blood samples of ID2 intensively, dotted ß-lactoglobulin only slightly. No IgE was detected by dotted human milk proteins, bovine and human α-lactalbumin (Multiplex Dot test; both boxes with a solid line and the dotted line).

Analyzing the BAT, the patient showed—before (ID2) (depicted in the upper BAT) and 5 months after delivery of her first child (ID2–1) (depicted in the lower BAT) —higher basophil activity against bovine milk, the single bovine caseins and ß-lactoglobulin, compared to human milk as well as human and bovine α-lactalbumin. 

For the severely allergic child ID3, with a positive oral challenge in early childhood, serum concentrations of total IgE and specific IgE to milk proteins were tested twice (ID3 in 2016) (CAP data with filled bars) and 2019 in parallel to the BAT (ID3–1) (CAP data with hatched bars). Whereas total IgE even increased from 2096 to >2500 IU/mL, specific IgE against milk protein decreased from 25.10 kU/L to 12.0 kU/L, as did IgE to bovine casein (from 19.5 kU/L to 12.8 kU/L), to bovine α-lactalbumin (from 11.8 kU/L to 2.34 kU/L), and to ß-lactoglobulin (from 0.61 kU/L to 0.31 kU/L). However, the sIgE concentration was highest against caseins (Figure 1-ID3).

In the dot test, the patient had intensive IgE reactivity against cow’s milk and α- and ß-casein and with less extent to κ-casein (Multiplex Dot test; boxes with a solid line). At the second sampling, the sensitization appeared to have become less strong. Also, the intensity of IgE against dotted bovine α-lactalbumin decreased (boxes with the dotted line). 

The BAT showed biological activity against all analytes exhibiting the highest basophil activity, when the basophils were incubated with caseins (ID3-BAT). 

### 3.3. Patients with Milder Reactions

**Figure 2:**
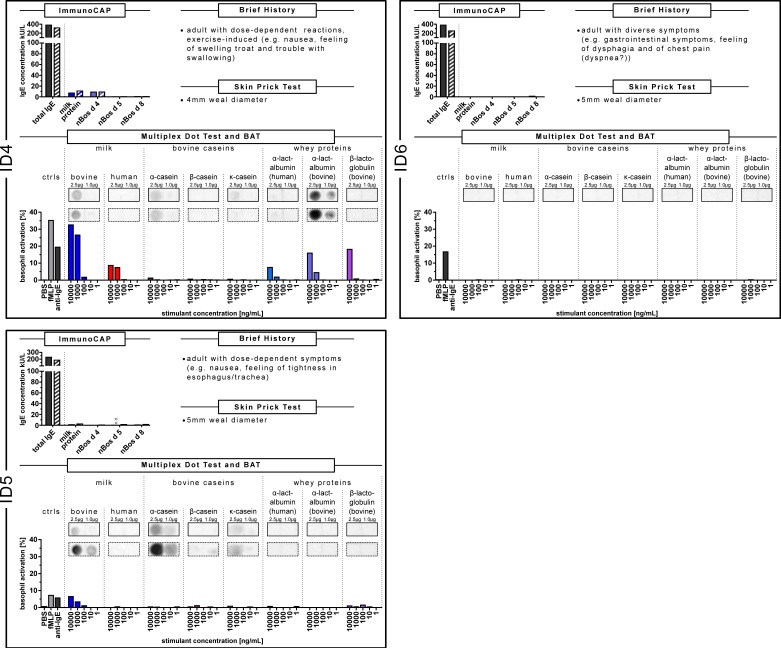
Synopsis of data of the sensitization pattern of ID4, ID5 and ID6 with milder allergic reactions in the ImmunoCAP, multiplex dot test and BAT. Whey proteins were more pronounced with this clinical response. CAP data of the CMA patients were depicted in a bar graph. The filled bars show the data of the first blood sample, the hatched bars are those of the second blood sample. Determination of IgE recognition to whole and single allergens of cow’s milk and human milk in the multiplex dot test. The concentration of the dot-blotted analytes as indicated was 1.0 µg/µL and 2.5 µg/µL, respectively. The IgE recognition of the first blood sample was given in a box with a solid line, of the second blood sample with a box with a dotted line. The BAT was performed with blood of the patients using whole cow’s milk and human milk and single bovine and human milk allergens. Percentages represent CD63 positive basophils determined by flow cytometry. Blood sample stimulation was conducted with the analytes as indicated. fMLP, anti-IgE were run as positive controls, PBS as negative control.

The subgroup of CMA patients with milder reactions, ID4, ID5 and ID6, showed a similar sensitization pattern in the CAP-FEIA, the multiplex dot test and BAT (comprised in Figure 2), 

ID4, with dose-dependent and exercise-induced symptoms after milk ingestion, showed the following CAP-FEIA sensitization after being tested on two different visits in our allergy outpatient clinic (in 2016 (ID4)) and 2019, parallel to the BAT (ID4–1) (Figure 2). Total IgE decreased slightly from 379 to 317 IU/mL, with minor changes in the ß-lactoglobulin-IgE-concentration from 0.34 kU/L to 0.56 kU/L. At any rate, this patient showed higher IgE-concentrations against the whey protein α-lactalbumin in comparison to casein (ID4-ImmunoCAP data, at the top left). 

In the dot test, the IgE reactivity against cow’s milk was less pronounced. The IgE reactivity was lower against the single caseins tested, compared to the stronger IgE reactivity against bovine α-lactalbumin (Multiplex Dot Test; both boxes with a solid line and the dotted line). Accordingly, the BAT revealed the highest reactivity against the whey proteins bovine ß-lactoglobulin, bovine and human α-lactalbumin and none against caseins (ID4-BAT).

For patient ID5, with nausea and feeling of tightness in the throat after milk ingestion, we measured a decrease of total IgE from 229 IU/mL to 185 IU/mL and an increase both in the IgE-concentration to caseins (from 0.70 kU/L to 1.57 kU/mL) and to α-lactalbumin (from 0.20 kU/L to 0.56 kU/L) at two visits, where blood samples were taken (ID5 and ID5–1 parallel to the BAT; Figure 2, ID5, ImmunoCAP data). In the dot test the IgE-reactions of the patient were less intense to the dotted milk allergens, but with an increasing IgE reactivity against α- and κ-casein (ID5 in comparison to ID 5–1; ID5-Multiplex Dot Test; boxes with a solid line compared to the dotted line). In the BAT, the basophils responded merely to cow’s milk and to a lower level to ß-lactoglobulin in comparison to the other stimulants (ID5-BAT).

In the CAP-FEIA analysis, ID6, with milder reactions to milk, showed higher concentrations of total IgE (385.0 IU/L) and specific IgE against casein (0.82 kU/L, next to negative specific IgE against the whey proteins α-lactalbumin and ß-lactoglobulin) within the first determination (ID6 in 2017) compared to less total IgE (251 IU/mL) and negative specific IgE against caseins in the second blood sample, parallel to the BAT (ID6–1; Figure 2; ID6-ImmunoCAP data with filled and hatched bars). Actually, all specific IgE concentrations against milk, milk products and single milk components became <0.01 kU/L between 2017 and 2019. No IgE reactivity could be determined in the dot test with the patient’s serum sample parallel to the BAT. Unfortunately, the first blood sample from 2017 was no longer available. In the BAT, ID6, however, was a non-responder (Figure 2, ID6-BAT).

ID7, as a non-allergic individual, did not show any specific IgE against milk and milk allergens in the ImmunoCAP, nor any IgE reactivity against the dotted milk allergens. In the BAT, the basophil activation was very low in comparison to the anti-IgE control (unlike ID1–ID5, in which at least one stimulant was equal or higher than the anti-IgE control) (data are shown in Supplemental Figure S1; the data of the Multiplex Dot Test of all patients with CMA, in comparison to the non-allergic individual, are additionally shown in Supplemental Figure S2). 

## 4. Discussion

In this study, we present comprehensive data of different diagnostic tests to characterize sensitization patterns of patients with different clinical pictures of CMA. The BAT and a newly developed multiplex dot test are novel tools beyond the current component-resolved diagnosis of CMA, evaluating the sensitization patterns for both cow’s milk and human milk and their single allergens. We found that diagnostic measures could be improved by this molecular characterization in our study group of six patients with different clinical patterns of CMA.

Our CMA patients reported severe and even life-threatening reactions already to traces of milk, so that the challenge tests for ID1 and ID2 were contra-indicated. Even skin prick testing would have been too risky for patients—such as patient ID1—with anaphylactic reactions to food allergens. 

The determination of allergen-specific IgE in CMA patients’ serum is an approved test to identify cow’s milk-sensitized patients. Accurate diagnosis of IgE-mediated CMA was improved by the introduction of the allergenic milk molecules caseins (Bos d 8), α-lactalbumin (Bos d 4) and ß-lactoglobulin (Bos d 5) [3]. Hence, for routine allergy diagnostic tests, total serum IgE, specific IgE to milk, cheese, milk from other species and these single milk allergens are available for CAP-FEIA. 

For our CMA patients we found that the anti-casein-IgE concentrations in patients ID1, ID2 and ID3 with a history of anaphylaxis or severe respiratory reactions were higher compared to the patients with less severe clinical reactions upon milk contact (ID4, ID5, ID6). In this respect, our study could discriminate between those patients who experienced severe symptoms that have persisted since childhood (ID1, ID2) and those who have had less severe clinical responses including exercise-induced symptoms (ID4). However, patient ID5 had an increase in specific IgE against casein, probably as a prognostic marker for an upcoming increasing clinical response to cow’s milk. On the contrary, ID3 showed a decrease of specific IgE against caseins between two visits, which might be an indication for a better tolerance to milk. Among others, Garcia-Ara [7] and Ito et al. [8] described that casein is a predictor for a prolonged CMA. These findings are in line with a recent study of Chatchatee et al. [16] confirming that IgE directed against the sequential casein epitopes predict persistent CMA. For the CMA patients of our study with less severe symptoms, our data were able to show lower IgE concentrations against caseins, which again support the above-mentioned literature. 

In earlier studies, IgE concentrations to the whey proteins α-lactalbumin (Bos d 4) and ß-lactoglobulin (Bos d 5) did not show a clear association with a certain degree of severity of CMA. As such, the assumption that higher specific IgE concentrations to whey allergens are characteristic for persistent CMA it is not clearly defined in the literature. In our study, however, we found that higher IgE antibody concentrations against whey proteins compared to caseins helped to identify those patients who were less severely affected by CMA. 

To date, the allergen-specific IgE detection via CAP-FEIA (namely sIgE against milk, against Bos d 4, Bos d 5 and Bos d 8) lacks allergens that became important for our CMA patients. In particular, patient ID2 with severe reactions upon contact to cow’s milk was concerned about a possible sensitization to human milk proteins and their clinical implications for her during breastfeeding. Apart from this peculiar clinical case, IgE-reactivity to human milk was described in patients with CMA [17,18]. Hence, we developed a component-resolved dot test for a more refined diagnosis of cow’s milk and human milk allergens. By this, with respect to cow’s milk, IgE reactivity was able to be differentiated into a broader sensitization pattern against whole cow’s milk, bovine α-, ß-, and κ-caseins, bovine ß-lactoglobulin and bovine α-lactalbumin. For human milk, IgE sensitization against whole human milk and human α-lactalbumin could be analyzed. To the best of our knowledge, this is the first component-resolved test for CMA diagnosis identifying milk allergen profiles thus detailed, as well as taking human milk proteins into consideration. 

Our data could show that the dot test in a more refined analysis was a suitable test to differentiate between CMA with a more severe clinical outcome (ID1, D2, ID3) derived from the binding intensity to the panel of tested allergens compared to the patients with less pronounced symptoms upon milk contact (ID4, ID5, ID6). Compared to the anaphylactic patients with CMA, patient ID5 (ID5–1) yielded a weaker IgE reactivity to cow’s milk, presumably representing a developing response to cow’s milk. Therefore, this patient should be closely monitored. Moreover, we found that patients ID1 and ID2 were highly reactive to the panel of bovine α-, ß-, and κ-caseins, whereas the patient ID3 depicted less IgE against κ-caseins, which might—discussed here with due caution—indicate that the patient becomes more tolerant to milk. Again interestingly, patient ID5 recognized bovine α-casein (ID5–1), evidently indicating an upcoming response to cow’s milk.

Remarkably, the human milk dot test proved to be slightly positive for patient ID1 with anaphylactic reactions to traces of cow’s milk, who reported on severe reactions as a breast-fed baby, despite strict cow’s milk avoidance of his nursing mother. On the other hand, the observation that no reactivity was evident for patient ID2 was particularly important for the anaphylactically reacting mother, who feared clinical reactions upon contact to human milk while breast feeding. 

For patient ID2, these findings were supported by negative IgE-concentration against human α-lactalbumin. With respect to the bovine whey proteins α-lactalbumin and ß-lactoglobulin, patients ID1 and ID3 with severe reactions to cow’s milk had positive IgE against α-lactalbumin and to a lesser extent also ID4. In contrast, ß-lactoglobulin appeared clearly predominant only for ID1. 

Whether the developed dot assay revealed superior diagnostic accuracy to the routine CAP-FEIA test when looking at the same allergens is difficult to assess. In fact, both serologic tests reveal comparable IgE sensitization patterns. Therefore, the multiplex dot test improves CMA diagnosis as the sensitization towards human milk proteins can be mirrored, which has to be kept in mind also in CMA patients, when studying patient ID2 [17,18]. 

Yet, both allergy diagnostic tests primarily identify the sensitization by means of the presence of allergen-specific IgE, not the clinical response. However, to assess the current clinical response to cow’s milk and also human milk allergens, we performed BAT with the blood of our CMA patients. Consistent with literature [19,20,21,22], the BAT pinpointed the clinical response of CMA best. Our results showed that the basophil activity is correlated with the degree of symptoms. In this respect, it is important that the allergen-induced basophil activation always has to be compared to the anti-IgE activation of the individual patient. In particular for our patients with a more alarming CMA history, the BAT revealed that the basophils were more reactive to allergen stimulation depicting the biological activity against the allergens tested. For ID1, basophil activation before OIT with adjunctive treatment with omalizumab was clearly shown upon stimulation with cow’s milk, in particular κ-casein and ß-lactoglobulin and lower against all the other allergens. Evidently, cow’s milk rather than human milk is more pronounced in the allergic response in our patient with severe cow’s milk allergy since early infancy. 

Remarkably, for patient ID2, our data could elucidate that the allergens tested induced basophil activation at lower allergen concentrations, meaning that less allergen is mandatory for CD63 activation. This is described by Hoffmann et al. [23] reporting on the basophil sensitivity (CDsens), defined as the allergen concentration, at which half of all reactive basophils respond. Also, patient ID3 has a high added value by means of the BAT to the sensitization profiles by dot test and CAP-FEIA determination depicting the activation of basophils after stimulation with all analytes and in low concentrations. In contrast, the BAT distinguished for ID4 and ID5 less severe sensitization status, for ID4, interestingly, a less symptomatic clinical outcome, as the patient merely represented a whey-sensitization. For ID5, our study revealed that the BAT is a suitable tool to monitor and detect changes in the in vitro immune response of his clinical milk reactivity, which is to be expected from the data of the CAP-FEIA and dot test.

However, our study group also demonstrated the limitations of BAT for CMA diagnosis. Patient ID6 represents a non-responder who did not show any CD63 activation through anti-IgE stimulation. Among others, Lötsch et al. [24] reported on 3.25–6.5% of non-responders dependent on the readout when studying BAT for hazelnut allergic patients. In such cases, the BAT was of no value; hence, the classical CAP-FEIA test and the multiplex dot test have merit and may provide reliable results.

Our study group assesses only a small number of CMA patients. However, we were able to differentiate between the various clinical pictures of CMA patients from severe anaphylactic forms identified as casein allergics to milder forms of CMA identified as whey protein allergics. 

The strength of this study is that both our multiplex dot assay and the BAT are superior to IgE recognition in routine diagnostic tests, and we were the first to show that both tests make more cow’s milk and human milk allergens accessible for CMA diagnosis. Thus, they enable us to show both the sensitization to cow’s milk as well as to human milk proteins. Finally, our BAT with its extensive and specific readout [13] helps to improve the diagnostic accuracy: (1) It mirrors the acute degree of CMA against cow’s milk and human milk allergens, and (2) provides monitoring of the development of CMA.

## Supplementary Materials

The following are available online at https://www.mdpi.com/2072-6643/11/6/1331/s1, Figure S1: Synopsis of data of the non-allergic individual ID7 in the ImmunoCAP, Multiplex Dot Test and BAT. Figure S2: Determination of IgE recognition of patients with CMA and a non-allergic individual in a Multiplex Dot Test. Table S1: Summary of data for patients with milk allergy.
