# Supplementary material for: Individual Sensitization Pattern Recognition to Cow’s Milk and Human Milk Differs for Various Clinical Manifestations of Milk Allergy
Source: Nutrients. 2019 Jun 14;11(6):1331. doi: 10.3390/nu11061331 (PMC6628265; doi:10.3390/nu11061331)
Supplement: Supplementary file 1 [file nutrients-11-01331-s001.pdf]

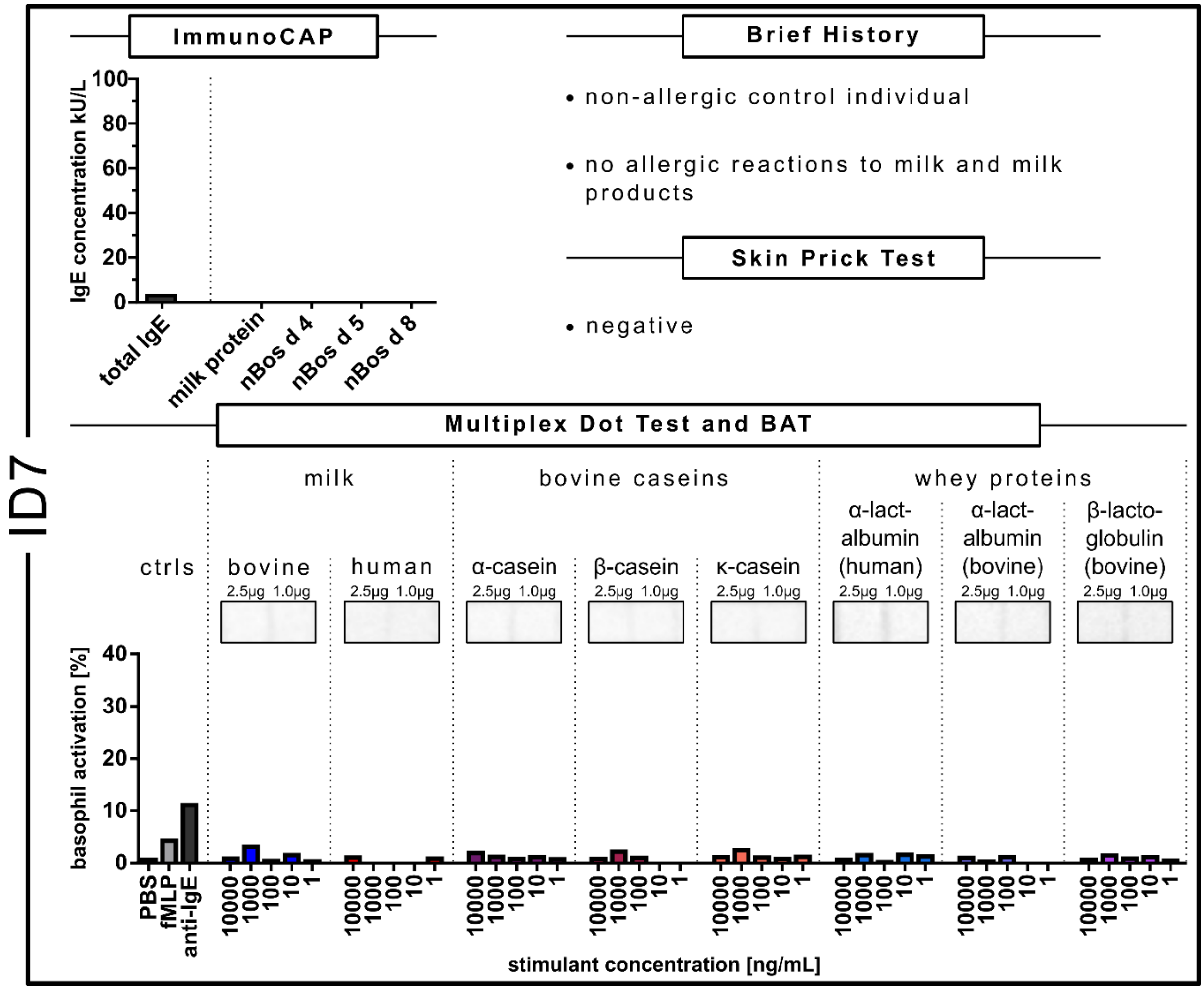

Supplemental Figure S1. Synopsis of data of the non-allergic individual ID7 in the ImmunoCAP, Multiplex Dot Test and BAT. CAP data of the negative control individual are depicted in a bar graph. Determination of IgE recognition to whole and single allergens of cow´s milk and human milk in the multiplex dot test. The concentration of the dot-blotted analytes as indicated was 1.0 µg/µL and 2.5 µg/µL respectively. The BAT was performed with blood of the non-allergic control individual using whole cow's milk and human milk and single bovine and human milk allergens. Percentages represent CD63 positive basophils determined by flow cytometric analyses. Blood sample stimulation was conducted with the analytes as indicated. fMLP, anti-IgE were run as positive controls, PBS as negative control.

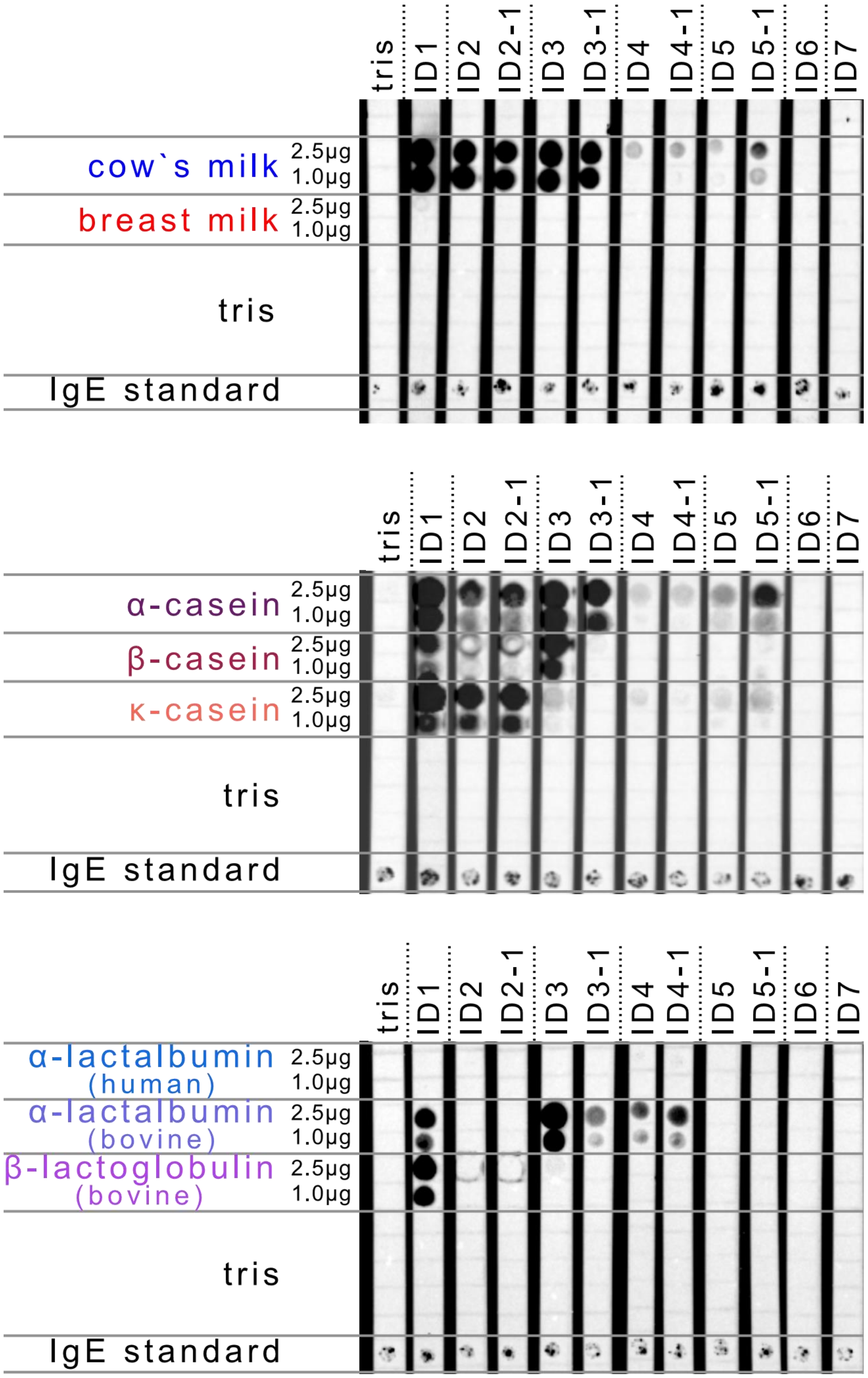

tris
ID1
ID2
ID2-1
ID3
ID3-1
ID4
ID4-1
ID5
ID5-1
ID6
ID7
cow`s milk
2.5μg
1.0μg
breast milk
2.5μg
1.0μg
tris
IgE standard
tris
ID1
ID2
ID2-1
ID3
ID3-1
ID4
ID4-1
ID5
ID5-1
ID6
ID7
α-casein
2.5μg
1.0μg
β-casein
2.5μg
1.0μg
κ-casein
2.5μg
1.0μg
tris
IgE standard
tris
ID1
ID2
ID2-1
ID3
ID3-1
ID4
ID4-1
ID5
ID5-1
ID6
ID7
α-lactalbumin (human)
2.5μg
1.0μg
α-lactalbumin (bovine)
2.5μg
1.0μg
β-lactoglobulin (bovine)
2.5μg
1.0μg
tris
IgE standard

Supplemental Figure S2. Determination of IgE recognition of patients with CMA and a non-allergic individual in a Multiplex Dot Test. The concentration of the dot-blotted analytes as indicated was 1.0 µg/µL and 2.5 µg/µL, respectively.

Supplemental Table S1 Summary of data for patients with milk allergy

Severtity of symptoms	Pat. ID	Sex	Age	SPT$_{Milk}$	IgE ImmunoCAP [kU/L]/CAP class													Symptoms to milk ingestion
						Bovine milk				Cheese and milk of other species					Single allergens			
					Total IgE [IU/mL]	Milk protein (f2)	Milk boiled (f231)	Whey (f236)	Cheddar cheese (f81)	Mould cheese (f82)	Ewe´s milk (f325)	Ewe`s whey (f326)	Goat´s milk (f300)	Mare´s milk (f286)	Bov. casein (nBos d 8)	ß-lacto-glob. (nBos d 5)	α-lactalb. (nBos d 4)	
Anaphylaxis/ Severe reactions	**ID1**	M	30	in early infancy; recently contra-indicated	85.0	34.90 (4)	38.00 (4)	33.60 (4)	29.80 (4)	23.70 (4)	30.60 (4)	25.80 (4)	32.00 (4)	2.55 (2)	31.30 (4)	8.86 (3)	8.85 (3)	**Anaphylactic reactions** since early childhood ((also to breast milk); cardioplegia after accidental intake of traces of milk. OIT since 2016.
	**ID2**	F	32	recently contra-indicated	115.7	1.32 (2)	1.24 (2)	0.91 (2)	1.26 (2)	0.63 (1)	0.79 (2)	0.77 (2)	0.81 (2)	negative (0)	1.27 (2)	negative (0)	negative (0)	Skin reactions since early childhood (even as breast-fed child). Self-guided oral desensitization in 2008/2009, thereafter more severe reactions (urticaria, flush, asthmatic reactions, at first exercise-induced). After milk avoidance **anaphylactic reactions** to minute amounts of milk.
	**ID2-1**				78.5	0.79 (2)	0.81 (2)	0.59 (1)	0.75 (2)	0.35 (1)	0.49 (1)	0.45 (1)	0.56 (1)	<0.01 (0)	0.72 (2)	0.18 (0)	0.17 (0)	
	**ID3**	F	9	n.a. after 20 min; no late reaction*	2096	25.10 (4)	29.20 (4)	25.10 (4)	n.d.	n.d.	n.d.	n.d.	n.d.	n.d.	19.50 (4)	0.61 (1)	11.8 (3)	Diagnosed milk allergy since 2010. **Severe reactions** at the skin and respiratory tract (cough, dyspnea)
	**ID3-1**				>2500	12.00 (3)	14.40 (3)	7.34 (3)	11.80 (3)	3.11 (2)	7.34 (3)	7.92 (3)	9.58 (3)	3.61 (3)	12.80 (3)	0.31 (0)	2.34 (2)	
No anaphylaxis	**ID4**	F	26	+	379.0	7.25 (3)	0.77 (2)	1.54 (2)	0.33 (0)	0.85 (2)	1.15 (2)	1.86 (2)	0.63 (1)	0.37 (1)	0.62 (1)	0.34 (0)	8.90 (3)	Diagnosed milk allergy since 2016. Reactions (nausea, feeling of swelling of throat and troubles with swallowing) primarily exercise-induced. Different amounts of milk and milk products can be tolerated.
	**ID4-1**				317.0	10.90 (3)	0.87 (2)	1.97 (2)	0.32 (0)	0.86 (2)	1.01 (2)	2.45 (2)	0.72 (2)	0.44 (1)	0.64 (1)	0.56 (1)	9.19 (3)	
	**ID5**	**M**	25	+	229.0	1.09 (2)	0.97 (2)	0.46 (1)	0.43 (1)	0.23 (0)	0.88 (2)	0.62 (1)	0.55 (1)	0.01 (0)	0.70 (1)	n.d.	0.20 (0)	Diagnosed milk allergy since 2016. Nausea, feeling of tightness in esophagus/trachea.
	**ID5-1**				185.0	2.47 (2)	1.87 (2)	0.85 (2)	0.90 (2)	0.40 (1)	1.67 (2)	1.18 (2)	1.24 (2)	<0.01 (0)	1.57 (2)	1.46 (2)	0.56 (1)	
	**ID6**	M	53	+	385.0	n.d.	n.d.	n.d.	n.d.	n.d.	n.d.	n.d.	n.d.	n.d.	0.82 (2)	negative (0))	negative (0)	Diagnosed milk allergy since 2000. Gastrointestinal symptoms, feeling of dysphagia and of chest pain (dyspnoe?)
	**ID6-1**				251.0	<0.01 (0)	<0.01 (0)	<0.01 (0)	<0.01 (0)	<0.01 (0)	<0.01 (0)	<0.01 (0)	<0.01 (0)	<0.01 (0)	<0.01 (0)	<0.01 (0)	<0.01 (0)	
**Negative control**	**ID7**	F	55	-	3.0	<0.01 (0)	<0.01 (0)	<0.01 (0)	<0.01 (0)	<0.01 (0)	<0.01 (0)	<0.01 (0)	<0.01 (0)	<0.01 (0)	<0.01 (0)	<0.01 (0)	<0.01 (0)	No reactions to milk and milk products.

SPT: Skin Prick Test; n.a. not available; n.d. not determined; *ID3 is a child with a food allergy triggered atopic eczema, which is why the late reaction to SPT was documented. This child was positive in a titrated oral challenge with cow's milk (urticaria); +. positive; -: negative

	Pat. ID	Other sensitizations**		Other allergic symptoms
		Aeroallergens	Foods	
Anaphylaxis/ Severe reactions	ID1	-	-	Allergic asthma; hazelnut, peanut sensitization in childhood
	ID2	Hazel, birch, grass pollen, HDM	Egg, peanut, hazelnut, fish, cashew, pistachio, seafood	Atopic dermatitis since infancy, allergic asthma since early childhood, allergic rhinoconjunctivitis, severe reactions (systematic reactions)
	ID3	n.d.	Peanut, egg, soy, hazelnut, almond, lupine, sunflower seeds	Allergic asthma, atopic dermatitis, severe reactions (systemic reactions)
No anaphyla-xis	ID4	rPhl p1, rPhl p 5 (grass pollen)	Egg, celery, hazelnut, apple, carrot, tomato	Allergic rhinoconjunctivitis, OAS
	ID5	Rye, birch (t3), rBet v 1 (t213), rPhl p1, rPhl p 5 (grass pollen), HDM	Egg, celery, hazelnut, apple, carrot, tomato	Allergic rhinoconjunctivitis, asthma
	ID6	Rye, birch, grass, hasel, HDM	none	Allergic rhinoconjunctivitis.
Negative control	ID7	none	none	none

n.d.: not determined; **: determined by SPT or IgE ImmunoCAP; OAS: Oral Allergy Syndrome; HDM: House dust mite
